# Supplementary material for: The interactome of intact mitochondria by cross-linking mass spectrometry provides evidence for coexisting respiratory supercomplexes
Source: Mol Cell Proteomics. 2017 Dec 8;17(2):216–32. doi: 10.1074/mcp.RA117.000470 (PMC5795388; doi:10.1074/mcp.RA117.000470)
Supplement: Supplemental Data [file supp_RA117.000470_133922_0_supp_23978_fzffwf.zip › spectra_annotation/mito_spectra_annotation/473-1-3-1-4-1.pdf]

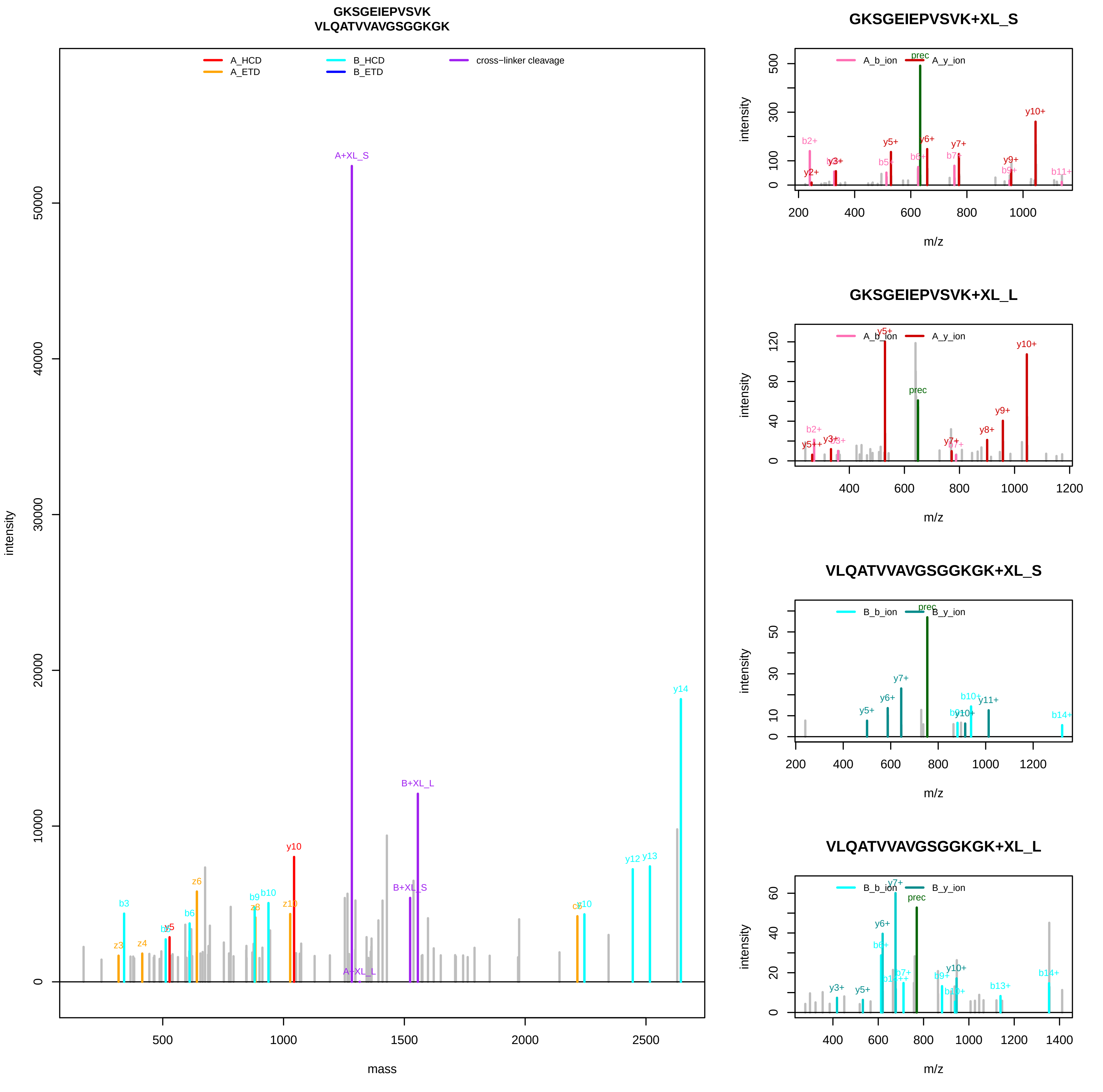
GKSGEIEPVSVK
VLQATVVAVGSGGKGK
A_HCD
A_ETD
B_HCD
B_ETD
cross−linker cleavage
A+XL_S
B+XL_L
B+XL_S
A+XL_L
y14
y12
y13
y10
z10
b10
b9
z8
z6
b6
b3
y5
z3
z4
y10
intensity
mass
GKSGEIEPVSVK+XL_S
A_b_ion
A_y_ion
prec
y10+
y5+
y6+
y7+
b2+
y9+
b11+
m/z
GKSGEIEPVSVK+XL_L
y10+
y9+
y8+
y7+
b2+
m/z
VLQATVVAVGSGGKGK+XL_S
B_b_ion
B_y_ion
y7+
y6+
y5+
b10+
y11+
b14+
m/z
VLQATVVAVGSGGKGK+XL_L
y7+
y6+
b6+
y3+
y5+
y10+
b13+
b14+
m/z
